# Non-dispensing pharmacists integrated into general practices as a new interprofessional model: a qualitative evaluation of general practitioners’ experiences and views

**DOI:** 10.1186/s12913-024-10703-y

**Published:** 2024-04-23

**Authors:** A.C.M. Hazen, V.M. Sloeserwij, E. de Groot, J.J. de Gier, N.J. de Wit, A.A. de Bont, D.L.M. Zwart

**Affiliations:** 1 Department of General Practice, Julius Center for Health Sciences and Primary Care, University Medical Center Utrecht (UMCU), Utrecht University, Universiteitsweg 100 3584 CG Utrecht. Postal address STR 6.131, P.O. Box 85500, 3508 GA Utrecht, The Netherlands; 2 Department of Pharmacotherapy, - Epidemiology and – Economics, University of Groningen, Antonius Deusinglaan 1, Building 3214, 9713 AV Groningen, The Netherlands; 3 Tilburg School of Social and Behavioral Sciences, Warandelaan 2, 5037 AB Tilburg, The Netherlands

**Keywords:** Interprofessional model, Non-dispensing pharmacist, Primary care, General practice, Interview study, Quality improvement

## Abstract

**Background:**

A new interprofessional model incorporating non-dispensing pharmacists in general practice teams can improve the quality of pharmaceutical care. However, results of the model are dependent on the context. Understanding when, why and how the model works may increase chances of successful broader implementation in other general practices. Earlier theories suggested that the results of the model are achieved by bringing pharmacotherapeutic knowledge into general practices. This mechanism may not be enough for successful implementation of the model. We wanted to understand better how establishing new interprofessional models in existing healthcare organisations takes place.

**Methods:**

An interview study, with a realist informed evaluation was conducted. This qualitative study was part of the Pharmacotherapy Optimisation through Integration of a Non-dispensing pharmacist in primary care Teams (POINT) project. We invited the general practitioners of the 9 general practices who (had) worked closely with a non-dispensing pharmacist for an interview. Interview data were analysed through discussions about the coding with the research team where themes were developed over time.

**Results:**

We interviewed 2 general practitioners in each general practice (18 interviews in total). In a context where general practitioners acknowledge the need for improvement and are willing to work with a non-dispensing pharmacist as a new team member, the following mechanisms are triggered. Non-dispensing pharmacists add new knowledge to current general practice. Through everyday talk (discursive actions) both general practitioners and non-dispensing pharmacists evolve in what they consider appropriate, legitimate and imaginable in their work situations. They align their professional identities.

**Conclusions:**

Not only the addition of new knowledge of non-dispensing pharmacist to the general practice team is crucial for the success of this interprofessional healthcare model, but also alignment of the general practitioners’ and non-dispensing pharmacists’ professional identities. This is essentially different from traditional pharmaceutical care models, in which pharmacists and GPs work in separate organisations. To induce the process of identity alignment, general practitioners need to acknowledge the need to improve the quality of pharmaceutical care interprofessionally. By acknowledging the aspect of interprofessionality, both general practitioners and non-dispensing pharmacists will explore and reflect on what they consider appropriate, legitimate and imaginable in carrying out their professional roles.

**Trial registration:**

The POINT project was pre-registered in The Netherlands National Trial Register, with Trial registration number NTR-4389.

**Supplementary Information:**

The online version contains supplementary material available at 10.1186/s12913-024-10703-y.

## Background

New models are emerging worldwide to organise and deliver pharmaceutical care. In Canada, Australia, the United Kingdom, Ireland and the Netherlands *non-dispensing clinical pharmacists* (NDPs) have been integrated in general practice teams, providing pharmaceutical care in close collaboration with the general practitioner (GP) [1–5]. This new interprofessional model appears to improve quality and safety of pharmaceutical care: in practices with fully integrated NDPs, drug therapy problems are adequately addressed and less medication-related hospitalisations occur [6–8]. 

It has been recognised that implementing promising interventions in a new context does not automatically improve the quality and safety of pharmaceutical care in the same way,, as their success can be highly dependent on the context in which the intervention is introduced [9]. Understanding the breadth of contextual influences are vital in conducting complex interventions [10] that require social interaction between professionals: such interventions could work well in one context, but not at all in another [11]. In addition to answering the question *whether* the interprofessional model improves quality of care with quantitative studies, we need to understand *how and why* this improvement works (so-called working mechanisms) and *when* (so-called context elements). Understanding how, why and when are concepts of realist evaluation that came about due to challenges in implementing interventions in other contexts [9] These understandings could help to better interpret results found so far and could increase chances of success with broader implementation of the model in other practice settings.

Earlier theories on how and why new interprofessional models in healthcare could improve quality of care show the importance of *the addition of new knowledge* to existing organisations [12–14]. This has also been recognised for the introduction of clinical pharmacists in general practice teams [15] However, establishing new interprofessional models in existing healthcare organisations is challenging and interprofessional collaboration is not self-evident [16]. Hence, addition of new professional knowledge alone may not be enough for successful implementation.

When we introduced the interprofessional model in the Netherlands in the Pharmacotherapy Optimisation through Integration of a Non-dispensing pharmacist integrated in primary care Teams (POINT) project [5], our initial programme theory was that the addition of new knowledge was key. These general practice pharmacists add specific knowledge about the pharmacotherapeutic treatment of elderly patients with polypharmacy and multimorbidity, who often have complex pharmaceutical care needs [17]. To make optimum use of this additional knowledge brought into the practices by NDPs, it was considered essential that the NDP had a patient-centred approach in applying pharmaceutical knowledge. NDPs were additionally trained in communication, consultation and clinical reasoning skills [17, 18]. In the present study, we challenge and refine our theory on *when, why and how* the interprofessional model of integrating pharmacists into general practice works, using a realist informed evaluation.

## Methods

### Setting

In the Netherlands, general practice is provided by a team, consisting of GPs, practice assistants and practice nurse(s). These general practice teams are increasingly located in multidisciplinary health centres, with other disciplines such as physiotherapists, dietitians, dentists and social services. A community pharmacy is often available on-site. Community pharmacists and GPs work together to ensure the safe use and timely dispensing of medication. They have structural pharmacotherapeutic consultation meetings. The level of collaboration between community pharmacists and GPs varies throughout the country.

Although already implemented in other countries, the interprofessional model with an NDP integrated in general practice teams is a novel approach in the Netherlands (box 1). In the POINT project, where this study was part of, outcomes of the model were measured in ten general practices [19]. The practices could take part in the POINT study when they were explicitly willing to host an NDP and to cooperate in the development and evaluation of the new role of NDP. The practices needed to have a consultation room available for the NDP and to provide the NDP access to the GPs’ electronic medical records.

After an induction period of three months, the NDPs worked full time in the practices from June 2014 until May 2015, while concurrently being trained in a 15-month Clinical Pharmacy Training Program based on interprofessional workplace learning, to develop skills in communication and clinical reasoning [18]. One NDP was unable to finish the training program. Of the remaining nine NDPs, five continued working as an NDP in the general practice after the intervention period.

**Table:** 

**Box 1. Description of the NDP in the POINT project**
During the POINT project, the NDPs were given integral responsibility for the pharmaceutical care in the practice.
They intervened *at the patient level*, performing clinical medication reviews with elderly patients who use multiple medications and holding individual consultations for patients with specific drug therapy problems; and *at the practice level*, organising quality improvement projects and educating GPs and staff members on pharmacotherapy. The general outline of how NDPs were expected to fulfil their role was pre-specified, but NDPs were encouraged to develop their role during the study and to tailor it to the practice’ needs.

The training and professional identity development of the NDPs were described earlier [18, 20]. Quantitative evaluations demonstrated that implementation of the NDPs in general practice teams resulted in improved quality and safety of pharmaceutical care: we found a lower risk of medication-related hospitalisations amongst elderly patients with polypharmacy, compared to usual care [8] and that NDPs identified and adequately addressed drug therapy problems [21].

### Methodological approach

To evaluate the interprofessional model with an NDP integrated in general practice, which can be considered a complex intervention, we chose a realist informed evaluation, to explain how and why an intervention works, for whom and under what circumstances [22]. Thoroughly focussing on the context contributes to a better understanding of the (social) intervention effects. The combination of the intervention and its specific context are then thought to trigger mechanisms, which in turn produce both intended and unintended outcomes.

Elements on context, mechanisms and outcomes were inferred from the interviews. Context-elements were defined as “actors or factors that are external to the intervention, present or occurring even if the intervention does not lead to an outcome, and which may have influence on the outcome” [23] and mechanism-elements as underlying processes or structures, usually hidden, which operate in particular contexts and generate outcomes [24]. Combining these elements, we formulated theories on when, why and how the interprofessional model of an NDP integrated in general practice improves pharmaceutical patient care– an outcome based on findings of earlier quantitative analyses in the POINT project [8, 21]. 

### Recruitment and data collection

We interviewed GPs from the practices where an NDP had worked during the POINT project. To guarantee information-rich interviews, we used ‘intensity sampling’ (selecting extreme cases to uncover unique understandings) and ‘snowball sampling’ (selecting participants through referrals from existing participants) methods: we invited the nine GPs who supervised the NDPs at the workplace (intensity sampling) and asked them which colleague GP (still) had a close working-relationship with the NDP in daily practice, and/or a distinct opinion on the NDP (snowball sampling) [25].

Semi-structured interviews were conducted between March and June 2018 by one researcher who had experience with doing interviews (VMS, a PhD student and GP trainee, who alternates between periods of doing research and following general practice training) accompanied by a 6th year medical student (AnH, FW). The researchers had the skills to build rapport and the interviewees were familiar with the POINT project as a whole. Each medical student joined 9 interviews. Before the interviews, senior researchers (EdG, DZ) provided advise to the medical students about carrying out interviews and provided feedback after the first interviews were carried out. Interviews lasted between 30 and 45 min and took place in a private room at the GPs’ practices. All interviews were audio-recorded with a Sony audio recording device that was available through our organisation and used by researchers within our department to carry out interview studies.Some interviews were transcribed verbatim by the medical students and some by an agency with whom we have made arrangements on privacy. Interviews were anonymised by removing personal identifiers and were saved by using an encryption key, which was kept secure by one of the lead investigators.

The topic guide used for the interviews consisted of open questions, and was inspired by the realist evaluation framework [26, 27]. Within realist evaluation, the aim is to gather insight into how people react to the intervention (understanding how, why and when outcomes come about), rather than evaluating opinions about the intervention. Participants were asked to describe their experiences of the intervention, rather than asked for their opinions about the NDP. The interviewees were participating in the overarching POINT project voluntarily and were well aware that their opinion, negative or positive, was essential for us as researchers. We assumed that power dynamics were not at play in this study as the interviewees were GPs and most of the researchers involved in this study were GPs as well. The topic guide was adjusted after a pilot interview with a GP, to ensure that each topic was properly highlighted (see Online Supplement S1 for the topic guide).

### Data analysis

We analysed the interviews by focussing on the context, mechanism and outcome. We used a combined deductive and inductive approach as coding was guided by the initial ideas on how the context and the intervention contributed to the outcomes (deductive) and followed by looking for new, additional mechanisms within our data set (inductive).

All interview transcripts were coded independently by two researchers (VMS, and a 6th year medical student AnH or FW), using NVivo version 11.13. In the inductive coding, first, the exact words or phrases that were used by the interviewees were used as codes. Then, a more interpretative approach was used to capture the essence of the codes. Codes were regularly discussed within the research team (VMS, EdG, DZ, AdB), resulting in refined coding and suggestions for the identification of additional codes and themes. Discrepancies and ambiguities in coding and interpretation were resolved in discussion. In these regular discussion meetings, the senior researcher in qualitative research (EdG) gave feedback. She was not familiar with the interviewees and could take a more distant position during coding and interpretation. With this iterative, cyclical analysis we identified elements on context and mechanisms that, combined with outcomes previously found in our quantitative analyses [8, 21] resulted in a refined and deepened programme theory. The SQUIRE checklist was followed while writing the manuscript [28]. 

## Results

In total, 18 GPs were interviewed, with a mean age of 50 and on average 19 years of work experience (Table 1).

**Table 1:** Baseline characteristics GPs

	GPs (*n* = 18)
**Female**	10
**Age (years)**	50 ± 9.2
**Working experience (years)**	18.8 ± 10.7
**GP practice in urban setting**	14
**Employment**	
Salaried	7
Partner in general practice partnership*	11

Data are presented as mean ± SD or number

Urban setting is defined as more than 100.000 inhabitants

* General practice with two or more owners

We conceptualised our interpretations of the interviews into context, mechanisms and outcome (Table 2) which we will discuss in more detail below.

**Table 2:** Overview of contexts, mechanisms and outcome

Context	Mechanism	Outcome
• GPs acknowledge the need to improve the quality of pharmaceutical care • GPs are willing to engage in this improvement	• NDP adds new knowledge to general practice • Professional identity alignment	• Improved quality of care

### GPs acknowledge the need to improve and are willing to engage

Two context elements were essential for the new interprofessional model. First, whether GPs acknowledged the need to improve the quality of pharmaceutical care both in their practices, and on a personal level. One GP described it as follows:

*‘’I felt unsafe and I feared the possibility of being brought into court because of prescribing errors. (…) And now, I feel better, I am much closer to delivering good pharmaceutical care instead of wondering: ‘what don’t I know, what do I know, lots of things are happening [with this patient] and I don’t know what is going on’. Pharmaceutical care is such a complex domain.*’’(Participant 8).

Second, whether GPs were willing to engage in this improvement, whilst general practice is becoming more complex. As one GP said:

*“[as a GP] you need to know of a lot. Especially as secondary care is increasingly transferred to primary care, it is all just becoming very specialised, so yes we do need extra knowledge.”* (Participant 2).

The need for improvement was further endorsed by the decline in quality of pharmaceutical care that was experienced by the GP after the NDP left their practice. Returning to the traditional model with only the community pharmacist, often instigated by a lack of financial reimbursement for the interprofessional model with the NDP by healthcare insurers, felt like “*it all collapsed in ruins*” (Participant 1).

### Aligning professional identities through discursive actions

Conforming with the initial programme theory, the GPs recognised that NDPs brought new knowledge into their clinical practice:

*“Through their background, [the NDP] provides depth, they can explain in detail on what the medicine does with the body, or interactions with other medicines, and I think… as a GP your pharmaceutical knowledge is more shallow.”* (Participant 3).

*“They [the NDP] linked that to their knowledge on medications, to taper medications or to find an alternative, so they of course have the knowledge that I as a GP have not.”* (Participant 1).

In addition to this anticipated mechanism (adding new knowledge to the general practice team) we found that over time GPs and NDPs changed their professional identities.

By working as an interprofessional team, the GPs and the NDPs better understood what the other found appropriate (fitting behaviour in a certain context),legitimate (conforming to principles they recognize to be valid) and imaginable (collectively considering a broad range of possibilities and solutions to address issues). An example was that GPs increasingly valued differences in work approaches: NDPs work pro-actively while GPs work mainly reactively. In daily practice, an NDP pro-actively invites patients to their clinic with potential health concerns due to the combination of medication that they are using and their comorbidities. The NDP works together with the patient to prevent medication harm. A GP on the other hand reacts to the current presenting complaint of the patient. Another example of the GP and NDP better understanding what the other found appropriate and legitimate was observed by a GP who noticed that the NDP, over time, increasingly pursued patient-centric approaches:

*“I think that a pharmacist really has to get used to being located in general practice. General practitioners are kind of strange people, doctors think differently. (…) You know, in the beginning you have to get used to that and then (…) what a real difference is, is whether you see a list with medications or whether you see the patient using them. (…) That is the translation from practice to the medications, and that is the translation a pharmacist needs to complete, mainly in the beginning.”* (Participant 7).

The person-centred approach was also reflected in the communication between the GP and NDP:

*I notice that we come to the point much faster and quickly identify what the important issues are (about the care for the patient). They (NDP) often preselects what they need to discuss (…). It is also the experience they have gained over time*. (Participant 5)

Alignment of identities took place through discursive actions: as the GPs and NDPs talked in the corridors or during short daily meetings, about specific patients, their context and pharmacotherapeutic considerations. Alignment took time and was highly supported by the communication and clinical reasoning skills acquired by the NDPs during the training program, as NDPs learned to transition from drug-centred to patient-centred care, facilitating the NDPs’ identity changes. One GP described:

*They (NDP) were quite good at communicating as a community pharmacist, but they also learned that in general practice communication has a slightly different allure. (…) That you connect with what moves people and not just fire your questions at someone. An ongoing conversation instead of a (…) barrage of questions. (Participant 6)*

Alignment of identities between GPs and NDPs occurred through everyday talk which does not occur with community pharmacists. Discursively aligning what is appropriate, legitimate and imaginable did not occur with pharmacists who work in another organisation. One GP illustrated how he experienced the discourse with the NDP and with the community pharmacist differently:

“*To collaborate with someone with both medical knowledge as well as pharmaceutical background, that results in a nice cooperation. I sometimes visit the community pharmacist but that is different. You still have… then it is often all about logistics, while with [NDP] you notice that they just do much more with patients and, well, they have a much more medical background.*” (Participant 8).

Another GP explained: *“Because [name of NDP] is situated in the general practice, I can very easily walk over to their desk or put a memo in their work agenda and vice versa. It’s very easy to make contact with each other.”* (Participant 7).

Providing shared care for specific and complex patient problems enabled different opportunities for aligning identities as both enable GPs and NDPs to recognise, acknowledge and utilise the others’ expertise. For specific care, such as patients with a single drug therapy problem,GPs entrusted patients to the NDP or consulted the NDP for advice. In these situations, the GP easily agreed with the NDP’s recommendations, without much discussion. For example, in a patient with persistent pain who was referred to the NDP by the GP, the GP stated:

“*you know, when [NDP] says that starting pregabalin is the best option now, then I think Oh, good idea, well, let’s do that; as I do trust them, yes.*” (Participant 9).

For complex care– patients with multimorbidity, polypharmacy and multiple pharmaceutical care problems– the GPs recognised the importance of combining both their own and the NDPs expertise. The GPs experienced the need for face-to-face collaborative care meetings with the NDP to optimise the pharmaceutical care of these patients, for example when jointly carrying out clinical medication reviews in elderly patients. One GP described the importance of interprofessional collaboration in discussing such complex clinical medication review outcomes:

“*Sometimes, you [as a GP] think, what else is going on there? And then I wonder… they [the NDP] are trained as a pharmacist, and they looks through certain glasses. And I look through slightly different glasses. I definitely think these glasses are complementary, but I feel that sometimes there is a need for my broader scope, […] to see the wider picture, not focusing* solely *on the medication. Sometimes it is priority to make sure someone can stay home, or has a good quality of life, rather than to control the blood pressure; there is more to life than a controlled blood pressure.”* (Participant 5).

As a consequence of frequent and successful joint care meetings between GPs and NDPs, the following mechanism occurred: GPs started to think and feel differently about sharing (part) of their responsibility with the NDPs, amid the complexity of patients’ care needs. The GPs gradually entrusted parts of the provided care to NDPs, but meanwhile remained convinced that they should be able to provide the NDP-led care themselves– even though the GP recognised they actually would not be able to, especially in the complexity of care:

*“They [NDP] are better at it [providing pharmaceutical care] than I am. But I, as a GP, should be able to do it, too. […] That is how it always has been. Whether it stays like that, I don’t know.”* (Participant 9).

*“No, I think I cannot do all that what they [NDPs] can. […] I think that I should be able to do clinical medication reviews, but I am not sure whether I would be able to do it that good, no.”* (Participant 6).

*“So, I wouldn’t be able to do the same [as the NDP], even if I could take the same amount of time for the patient, because I do not have the knowledge.”* (Participant 1).

### Improved quality of care

The main outcome of the intervention was improved quality of care. A new, interprofessional model of pharmaceutical care, in which GPs and NDPs work closely together, through different mechanisms resulted in both perceived improvement of pharmaceutical care, as well as, as we have shown in our other studies, objectively demonstrated improvement outcomes [8, 21]. The newly formed model is not just another model of providing pharmaceutical care, but one in which interprofessional collaboration between GP and NDP becomes well established, as recognised by this GP:

“*Together, they make a very strong team.”* (Participant 1).

## Discussion

Our findings indicate that in a context where GPs acknowledge the need for improvement and are willing to engage in this improvement, working mechanisms are triggered. NDPs add new knowledge to current general practice. Through discursive actions both GPs and NDPs change in what they consider appropriate, legitimate and imaginable in their work situations. They align their professional identities. This contributes to the formation of an interprofessional healthcare model, in which shared care is provided by GPs and NDPs, resulting in improved quality of care.

Especially as the number of elderly patients with chronic conditions rises, GPs generally recognise that general practice requires a more diverse skill mix, and pharmacists’ expertise is suggested to be of additional value here [29, 30]. Although all GPs acknowledged there was a need for improvement of the pharmaceutical care provided in their practices, some GPs considered lack of time to provide pharmaceutical care as the main problem. Other GPs acknowledged that a gap in their own knowledge may hinder the provision of better pharmaceutical care. In our model, this element of the context clarifies whether the mechanism “willing to share their responsibility with NDPs” occurred. GPs who are acknowledging a personal knowledge gap seem more willing to share their responsibility with NDPs as opposed to GPs who only acknowledge a need for improvement on a practice level (related to lack of time). Recognising this difference between the contexts might be key for the implemented intervention to result in success [31]. 

When designing the interprofessional healthcare model, we assumed the addition of new knowledge by the NDPs to general practice to be a key working mechanism [17]– in line with previous theories [12–14]. A previous UK study, investigating stakeholder experiences of this interprofessional model with NDPs integrated into general practices, also found GPs appreciating the additional knowledge brought by NDPs [15]. However, additional knowledge is not always optimally utilised, because of existing boundaries between and within health professions that can lead to interprofessional conflicts [16, 32]. Ryan and colleagues describe challenges in realising effective interprofessional collaboration [15]. In their study, a GP compared the “perceived threat to professional boundaries and identity to that observed during the introduction of nurse practitioners, although suggested that this sentiment might be stronger since *everything a nurse can do a GP can probably do, whereas anything a pharmacist can do the GP probably can’t*” [15, p. 8]. Perhaps this limited degree of overlap between the two professions (GP and NDP) explains that professional boundaries between GPs and NDPs needed to be redrawn, and (interprofessional) identity work was needed.

A professional identity can be defined in terms of ‘spaces of action’, which are “what professional actors find appropriate, legitimate and imaginable in their work situations, given the existing cultural conditions” [33]. Spaces of action are not fixed. New spaces of action can be co-constructed and boundaries between interprofessional spaces of action can be redrawn, as spaces of action are the result of everyday work interactions, so-called discursive actions. In these discursive actions, the professional identities of both GPs and NDPs started to change. This process of aligning professional identities took place both explicitly and implicitly: GPs and NDPs became aware of what the other considers appropriate, legitimate and imaginable and started sharing ideas about these considerations, thereby re-drawing and aligning their professional identities.

We found that discursive actions were the means for the identity aligning process to take place; for example: knocking on each other’s door for ad hoc consultations during the day, coffee break meetings, asking the other to shortly pop over during a patient consultation to assess the patients’ pharmacotherapy directly together, or quick questions via digital notes in the patient records system. During these interactions, GPs and NDPs discussed specific patients and their context or pharmacotherapeutic considerations, thereby questioning each other’s routine, asking questions like “why do you do what you do?”. These discursive actions made GPs and NDPs both explicitly and implicitly reconsider what they thought appropriate, legitimate and imaginable in their work situations: the identity aligning process could take place, allowing for effective interprofessional collaboration.

Earlier studies already recognised ‘proximity’ between GPs and pharmacists, and them both working ‘on-site’ as important elements to enable interprofessional collaboration [15, 34]. A Canadian study on GPs’ experiences of prescribing pharmacists (both community pharmacists and NDPs, the latter being described as ‘team pharmacists’) reported that “the proximity of team pharmacists allowed physicians to develop trust and mutual respect with pharmacists; however, proximity alone did not facilitate collaboration.…All participants were hesitant to trust pharmacists with whom they were unfamiliar, especially in community settings.” [34, p.92] Besides the need for proximity, our study stressed that GPs and pharmacists need to be familiar with each other, i.e. work collaboratively to learn to speak the same language. Another study, in the United Kingdom, reported that “a strong preference was expressed [by GPs] for the pharmacy team to be located *in house all day*. In practices where the pharmacy team was located on-site, participants reported easy personal access and the ability to ask informal questions.” [15] That same study reported that ”where the pharmacy team was located off-site, however, they were viewed as *a separate entity* and aspects of communication were lost.” [15] We agree with those studies that proximity and working on-site are important, but we think that in this proximity GPs and NDPs not only need to get familiar but need to *align their professional identities*, which, in our view, incorporates deeper underlying mechanisms taking place than simply getting to know each other: it requires both parties to change. We believe that proximity and working on-site describe the essential conditions that are needed for this alignment process to take place: they allow for discursive actions to occur. ‘GPs in our study reported a difference between collaboration with NDPs and collaboration with community pharmacists, so, we hypothesise that despite (frequent) proximity between GPs and community pharmacists and (frequent) mutual relationships, community pharmacists and GPs may not have *aligned their professional identities*, whereas NDPs and GPs may have.

Over time, in the process of aligning identities, GPs may start to reconsider responsibilities. GPs feel responsible for the integral care provided to patients, including pharmaceutical care. This seems to be a core aspect of the GPs’ professional identity. For GPs, reconsidering responsibilities with other professionals is a delicate balance. On the one hand, the GP wants to be responsible for and in control of patient care, as becomes clear in the following quote of a Canadian GP: “*If they [pharmacists] are going to make clinical decisions about a patient, and they [pharmacists] don’t call me [to get my consent], that’s inappropriate”.* [34, p.91] On the other hand, the same GP had less problems with an NDP making clinical decisions as confirmed by the following quote: *“[The NDP] did not need to seek approval prior to prescribing whereas community pharmacists should.”* [34, p.91] It is possible to adjust the GPs identity by aligning with the NDPs’ professional identity. Our results indicate that mutual interactions between GPs and NDPs is an essential first step.

### Strengths and limitations

While many evaluations of interventions ignore the context in which the intervention is implemented, we instead aimed to better understand this context by using a realist informed approach. Taking the context into account provided additional insight into what could help to successfully implementat the interprofessional model with an NDP in other general practices.

This study had several limitations. Firstly, due to logistic reasons there was quite a large time frame between the ending of the intervention period (May 2015) and the interviews (March until June 2018). During this time, in five of the nine practices the NDPs continued working after the intervention period ended, while in the rest of the practices the NDPs stopped working, bringing different experiences to the fore. Secondly, there was little opposition to NDPs amongst the interviewees. This might be related to the selection of GPs and practices willing to engage to begin with. This limits possibilities to mirror different contexts. Lastly, we chose to include GPs only, for feasibility reasons. To obtain a broader overview of the context in which the NDPs were integrated, insights in the perspective of the full general practice team, including practice assistants and nurses would have added value.

### Implications for future pharmaceutical care and future research

The process of professional identity alignment is essential to make the interprofessional healthcare model work. It is important to highlight that this process is difficult, for both GPs and NDPs, and that it takes time. Understanding how the process takes place could help to optimise broader implementation of our interprofessional healthcare model.

Future follow-up research should investigate the health innovation sustainability of this interprofessional model on quality of pharmacotherapy in general practice, With the introduction of pharmacist prescribers in other countries like the United Kingdom, this may also be important with regards to roles and responsibilities of an NDP in general practice. Future research is needed to evaluate how and when this advanced role fits within the interprofessional model with an NDP. Also, the perspectives on the model of other stakeholders, such as policy makers, governmental bodies, professional organisations and healthcare insurers needs further investigation. We suggest that the relationship between financial sustainability and the importance of social factors (e.g. social interaction between professionals) should be the focus of future research too.

The need for additional training of pharmacists to work in general practice was recognised before [15, 35], yet we would like to specifically stress the importance of additional *interprofessional* training to further facilitate the process of identity alignment. Interprofessional training is a collaborative educational approach that involves both GPs and NDPs, working together to learn with, from and about each other. This training aims to improve teamwork, communication and the quality of patient care by fostering a deeper understanding of each other’s professional roles and responsibilities. It assists GPs and NDPs to develop the skills needed to work together effectively in a person-centred healthcare environment. We used a work-place based learning approach to develop these skills through practical experience and on-the-job activities [18]. In literature on interprofessional teams it was recognised that professional identity formation is a social activity, and professional identities are explored in relation with others [36]. Interprofessional training, like workplace learning, offer ample opportunities for informal conversations and reflections that will accelerate the process of identity alignment [37, 38]. 

## Conclusion

The new interprofessional healthcare model with the NDP integrated in general practice teams not only works through addition of new knowledge in general practice, but also via NDPs and GPs aligning their professional identities. This is essentially different from traditional pharmaceutical care models, in which pharmacists and GPs work in separate organisations. When broader implementation of the interprofessional model with NDPs in general practice is sought, GPs need to acknowledge that the need for improvement of the quality of pharmaceutical care requires focussing on interprofessional teamwork. Then, both GPs and NDPs will explore and reflect on what they consider appropriate, legitimate and imaginable in carrying out their professional roles for collaboratively providing the best pharmacotherapy to their patients.

### Electronic supplementary material

Below is the link to the electronic supplementary material.

Supplementary Material 1

Supplementary Material 2

## Data Availability

The datasets used and/or analysed during the current study available from the corresponding author on reasonable request.

## Abbreviations

NDP: Non-dispensing pharmacist
GP: General practitioner
POINT: Pharmacotherapy optimisation through integration of a non-dispensing pharmacist integrated in primary care teams
RE: Realist evaluation
