# Supplementary material for: Non-dispensing pharmacists integrated into general practices as a new interprofessional model: a qualitative evaluation of general practitioners’ experiences and views
Source: BMC Health Serv Res. 2024 Apr 23;24:502. doi: 10.1186/s12913-024-10703-y (PMC11040768; doi:10.1186/s12913-024-10703-y)
Supplement: Supplementary file 1 — Supplementary Material 1 [file 12913_2024_10703_MOESM1_ESM.docx]

**Online Supplement S1 – Topic guide used for the semi-structured interviews**

1. Fidelity of the intervention and the context in which the intervention was implemented.

Q1.1: Could you describe how pharmaceutical care is currently organised in your practice?

Q1.2: Please describe what was changed in this organisation by the arrival of the NDP? Either positively or negatively.

The NDP was "designed" to be a healthcare provider on the patient level (performing medication reviews, holding consultation hours) and on the practice level (doing quality improvement projects, educating practice staff). During the intervention year, the NPD and practices were allowed to tailor the NDP's role to the practice needs. Could you tell more about the NDP in your specific practice?

Q1.3: Do you have some examples of care problems where the NDP got/still gets involved?

2. How does the NDP work in this practice, for this organisation

Q2.1: What changed after the intervention period ended (and the NDP left your practice)?

Q2.2: Could you describe your collaboration with the community pharmacist, compared with collaborating with the NDP? How is this collaboration now, after the study, compared to the situation before?

Q2.3: Please describe pharmaceutical care that is provided by you, as a GP, compared to pharmaceutical care provided by the NDP? Are there differences in approaches, in care plans?

Q2.4: IF THE NDP STOPPED:
If there were no constraints by time or money, how should pharmaceutical care in your practice look like if it was up to you?
IF THE NDP CONTINUED:
this question is transferred

3. Sustainability: the future of the NDP in primary care

Q3.1: IF THE NDP STOPPED:
After the intervention period ended, the NDP left your practice. Can you describe the backgrounds thereof, illustrate how that went?
IF THE NDP CONTINUED:
After the intervention period ended, your practice started to employ the NDP. Can you describe the backgrounds thereof, illustrate how that went?

Q3.2: IF THE NDP STOPPED:
What would be needed for your practice to employ an NDP? Or more broadly: to optimise pharmaceutical care? Are there specific elements hampering improvements, or facilitating them?
IF THE NDP CONTINUED:
If there were no constraints by time or money, how should pharmaceutical care in your practice look like if it was up to you? Are there specific elements hampering improvements, or facilitating them?

4. Outcome: the additional value of an NDP in this practice, in this organisation

Q4.1: Could you describe what the NDP brought to your practice? Did all of the staff perceive the integration of the NDP in the same way, or were there differences?

Closing:

Q5.1: Is there anything else that you want to discuss that we didn't cover yet?

Thank the interviewee for his or her participation and time and stop recording.
