# Supplementary material for: Non-dispensing pharmacists integrated into general practices as a new interprofessional model: a qualitative evaluation of general practitioners’ experiences and views
Source: BMC Health Serv Res. 2024 Apr 23;24:502. doi: 10.1186/s12913-024-10703-y (PMC11040768; doi:10.1186/s12913-024-10703-y)
Supplement: Supplementary file 2 — Supplementary Material 2 [file 12913_2024_10703_MOESM2_ESM.pdf]

Reviewers’ reports

Reviews to Version 1:

Reviewer 1

Submitted: 27 Sep 2023

Feedback for the author(s)

Thank you for giving me the opportunity to review this article. This is a really interesting piece of work. Although there is now ample of literature on the topic of general practice-based pharmacists, the main concept of this 'realistic' study is novel and focuses on how professional identifies might be (re)shaped and thus interprofessional tensions overcome, as experienced/perceived by general practitioners. As authors rightly note, bringing new knowledge to general practices might not be enough to ensure successful integration of pharmacists and the potential for frictions/tensions between different professionals cannot completely be ruled out. This work might well be useful for policy attempting to introduce or shape general practice-based pharmacist services. The use of the Realist Paradigm ensures that important 'why' and in 'what context' questions are answered, thus translating findings into practical recommendations that can easily be employed by policymakers and clinicians.

I hope my comments below are of some use. My comments/suggestions mainly focus on better fitting the paper in the greater picture and improving the clarity of arguments (bearing in mind the international readership of BMC HSR). The use of English language was slightly problematic at times. Sentences and arguments were not always linked with each other, and arguments were often presented in isolation. I tried to make some changes where I could, but I believe that having a native English speaker proofreading the article would very much improve the clarity of presented arguments and would make the article appropriate for an international audience.

Look forward to seeing this article published!

Title:

-I suggest authors amend the title to indicate participants and study design, for example, "Non-dispensing pharmacists integrated into general practices as a new interprofessional model: a qualitative evaluation of general practitioners' experiences and views" or something along those lines.

Abstract:

-Lines 23-24: The term 'primary care' is slightly vague and means different things in different nations. Bearing in mind the international audience of BMC HSR, I suggest that authors use 'general practice' instead throughout the manuscript as a more self-explanatory term at a global scale.

-Line 25: '...in which they are implemented', please re-phrase this sentence as not currently clear what does 'they' refer to.

- Line 27: 'current practices', do authors refer to work practices? Or to general practices? Please clarify in the text.

-Lines 27-28: '...the addition of new professional knowledge alone may not be enough for successful implementation', this sentence does not necessarily convey the gap in knowledge that authors

address, in a few words please better clarify what is the gap in knowledge authors are trying to address.

-Line 32: 'We interviewed 18 general practitioners ', statements like such are rather 'Results' than 'Methods', as authors would not have known the precise number of participants beforehand. In the Methods, it is preferable to state how many GPs were invited instead (i.e., the available source of participants).

-Line 33: 'iterative, cyclic processes ', this is not a commonly used term necessarily, so, please define in a few words what does it mean.

-Lines 34-38: Results are composed of a single sentence, which is really long and very difficult to follow. Please consider splitting it into smaller sentences.

-Line 35: 'engage in this development', which 'development' are the authors referring to? Unclear to the readers currently.

-Line 35: 'working mechanisms ', need to define what this means as the word 'working' is a vague and bizarre term for readers without any experience of realistic perspectives.

Background:

-Line 53: change the first sentence to 'New models are emerging worldwide to organise and deliver pharmaceutical care'.

- Line 60: 'provide the same results', same results to what? Please consider re-phrasing.

- Line 61: 'This holds especially in case', suggest you change to 'Influence by the context especially applies to cases of complex interventions...'.

-Lines 64-65: explain to readers that 'how', 'why' and 'when' are all concepts of realism.

-Line 80: 'when, why and how the interprofessional model works', suggest you change to 'when, why and how the interprofessional model of integrating pharmacists into general practices works' or to something along those lines , so to clearly indicate what this interprofessional model is about.

Methods:

-Lines 86-87: Are GPs, practice assistants and practice nurse(s) indeed co-located in Dutch community pharmacies? If not, please re-phrase this sentence.

-Line 87: What sort of collaboration exists between GPs and community pharmacies in Netherlands under normal circumstances (i.e., when no NDPs are employed)? Would be interesting to clarify for readers outside Netherlands.

-Lines 91-92: 'The practices were selected', 'selected' in what terms? E.g. selected for integrating NDPs, selected for studying outcomes? Please clarify.

-Line 95: suggest you change 'introduction period' to 'induction period'.

-Box 1 is not cited at all within main text. Either cite it in text and guide readers accordingly or remove the box and have the content as a separate paragraph.

-Lines 110-114: I do not think that this paragraph has anything to do with 'Methods', perhaps it will fit better somewhere in the 'Background' section.

- Line 118: I suggest some caution with calling this study 'an evaluation'. For a study to be classified as 'evaluation', it has to fulfil very certain requirements. Either define 'evaluation' and explain why this study is considered an 'evaluation' or dismiss references to 'evaluation' completely (i.e., can simply refer to the study as 'study').

-Lines 120-121: '... as a social intervention alters and can be altered by the context in which it is introduced', I suggest you re-phrase this sentence as the second part of the sentence just repeats the same argument twice.

-Line 135: I suggest you define 'intensity sampling' and 'snowball sampling' in a few words, as these might not be self-explanatory terms for all readers.

-Line 138: 'In total, 18 GPs were interviewed', these statements are 'Results' rather than 'Methods', as you wouldn't have known this number beforehand (i.e., before study completion). Suggest you move to 'Results'.

-Lines 139-141: Were the interviews 'one-to-one'? Or there were 3 researchers (VMS, AnH and FW) present in each one? Will need to clarify.

-Lines 139-141: What experience/training the researchers had in qualitative research? Will need to clarify, especially if AnH and FW (medical students) carried out interviews on their own.

-Line 142: Where exactly within GP practices the interviews took place? Was privacy secured for all participants so to encourage expression of honest views? Please clarify.

-Lines 141-142: Did interviews complete when participants did not have anything else to share (i.e., in-depth interviews)? Or interviews would have stopped anyway should 45 minutes reached?

-Lines 142-143: What equipment was used for audio-recording interviews, who transcribed audio-records and how transcripts were anonymised? These details will need to be added.

-Lines 144-148: I suggest you change 'topic list' to 'topic guide'.

-Line 144: I suggest you spell out 'RE' and you clarify in a few words how exactly the topic guide was influenced by realism.

-Line 145: ' For this, participants were asked to describe their experiences with the intervention...', please explain what do you mean with 'this' (as currently not clear) and change 'experiences with' to 'experiences of'.

-How was rapport and trust built between researchers and interviewees? You will need to explain.

-Lines 146-147: I suggest you remove 'where necessary' (as it complicates the sentence) and I also suggest to explain how topic guide was piloted (e.g., on GPs?, NDPs?, researchers?).

-Line 153: ' First, codes were given that were close to the text', what does this sentence mean? It is totally unclear what 'close to the text' mean. Suggest re-phrasing.

-Was coding inductive or deductive?

-Lines 154-155: '... resulting in refined coding and suggestions for the identification of additional codes and themes', so, were all codes based on data exclusively? Were there any analytical themes derived? In general, how realism informed data analysis, e.g., were themes based on data and just presented in light of realism, or realism influenced the structure and synthesis of themes? Some more detail will be helpful for readers in here.

-Lines 158-159: 'Results were repeatedly compared to existing literature, to enhance credibility of the findings', I am totally confused with this sentence. How was credibility enhanced and in what terms? Say, for example, that results contradicted literature, would the authors have dismissed these results? Comparing findings with literature is always a required part in the 'Discussion' section, but not sure at all if and how this enhances credibility.

-How was reflexivity ensured in coding and data interpretation (if at all)?

Results:

-It would be interesting to start this section by presenting some key demographics of interviewees.

-Line 174: I would suggest you make the title of the theme more specific, e.g., Care Improvement.

-Line 177: change 'Next' to 'Second'.

-Lines 177-178: 'Primary care is getting more complex but GPs need to feel room for change and willing to engage', this sentence just repeats the quote. Quotes in qualitative research should provide data/new knowledge themselves. I suggest you remove this sentence.

-Line 178: Change 'put it' to 'said'.

-Lines 182-183: '...by the experienced decline in quality of pharmaceutical care...', this section of the sentence does not make sense, please consider re-phrasing. Not clear at all what 'experienced decline' means.

-Line 188: 'Conform' should be followed by 'with' or 'to', e.g., 'Conforming with the initial...'.

-Line 181, and throughout: suggest you spell out 'PR', e.g., Participant 2. As all participants were GPs, no need to state GP1, GP2, etc. after each quote.

-Line 199 and throughout: Unless journal guidelines instruct something different, it is usually a good practice to change pronouns 'he, his, him' and 'she, her' in quotes to 'they', 'their', 'them' etc. to minimise changes of participants being recognised through their quotes.

-Line 195: 'In addition to this anticipated mechanism...', re-state in a few words in brackets what the anticipated mechanism is.

-Line 199: ' NDPs work pro-actively while GPs work mainly reactively', any examples mentioned by participants? Would be interesting for readers.

-Line 199: 'Another example of this', what does this 'this' refer to? Unclear to readers.

-Lines 200-201: '... the NDP over time increasingly incorporated the context of the patient in their evidence-based considerations', this is a complicated and rather unclear section of the sentence. I suggest re-phrasing to '... NDPs, over time, increasingly pursued patient-centric approaches', and that way you also avoid repeating the quote in the narrative.

-Line 214: 'appropriate, legitimate and thinkable ', I am not sure at all that readers will understand the meaning of these terms, especially 'thinkable' is such a vague term. Please consider defining, somehow.

-Line 222: 'Providing shared care for specific and complex care ', suggest you change to 'providing shared care for specific and complex patient problems'.

- Lines 233-234: 'This was common in care for elderly patients with polypharmacy, for whom clinical medication reviews were performed', I cannot necessarily see what this sentence adds. You can

perhaps remove this sentence and add 'elderly' in line 230 where you list examples of patients. In any case, if you decide to keep this sentence, will need to define 'clinical medication reviews' and also explain where does 'This was common' refer to (i.e., what was common)?

-Lines 244-248: this paragraph should be re-phrased as it just re-iterates the quotes below. One suggestion might be to just keep the first sentence by re-phrasing it to: ' As a consequence of frequent and successful joint care meetings between GPs and NDPs, the following mechanism occurred: GPs started to think and feel differently about sharing (part) of their responsibility with the NDPs, amid the complexification of patients' care needs'.

-Lines 257-263: I cannot really see how the 'Improved quality of care' theme is different to 'Improvement' (the first theme). To me, both themes are talking about the same thing. In qualitative research, themes should ideally be mutually exclusive (i.e., no or limited overlapping content amongst different themes).

-Authors only present a few quotes from only 8 participants, with four quotes originating from the same participant (PR1). In qualitative reports, it is a good practice to try and give a say to as many participants as possible, rather than presenting multiple quotes from same participant. I would suggest including a few additional quotes from some other participants too.

-Overall, I believe that the Results section would benefit from a better/higher quality synthesis of data to produce mutually exclusive themes, so that content reflects the main concept/title of the theme and arguments flow naturally. Certainly, 18 qualitative interviews (each lasting 30-45 minutes) would have produced a rich dataset. At the moment, only a few ideas are presented, and often re-iterated under different themes (unfortunately).

-Contexts, mechanisms, and outcomes are not always clear and are somehow lost in the text, as often arguments are presented in isolation. I believe that should themes are refined, the addition of a Table/Figure summarising the identified contexts, mechanisms and outcomes (in a C+M=O formula) will benefit readers and raise the quality of data presentation.

Discussion:

-Lines 266-269: this sentence is really large and difficult to follow, please consider splitting into two smaller ones.

-Line 266: Start sentence by saying 'Our findings indicate that ...', or something along those lines, as at the moment it is not clear that arguments are based on this study's findings.

-Lines 274-275: I am really struggling to follow the following sentence 'Yet, we found some differences between GPs in our study in acknowledging the level of the problem at hand'. What sort of differences are authors referring to? Differences in opinions amongst GPs in the study? Which 'problem' are you referring to? What does 'at hand' mean? I suggest re-phrasing.

-Lines 275-279: this sentence is massive and unclear, strongly suggest that you split into two smaller ones. For example, '... recognising a lack of time to provide pharmaceutical care as the main problem...', what 'problem' are you referring to? This bit doesn't quite fit in the sentence. In addition, '...while others incorporated their own personal level in the problem too', how can you incorporate 'knowledge' into a 'problem', does not make sense, and it is unclear what 'problem' refers to.

-Lines 285-287: I suggest you simplify this sentence to 'A previous UK study, investigating stakeholder experiences of this interprofessional model with NDPs integrated into general practices, also found GPs appreciating the additional knowledge brought by NDPs '.

-Line 293: At the current reference list, Ryan et al. reference is number 15, not number 17.

-Line 293: 'Perhaps this explains', what does 'this' refer to? Please explain.

-Lines 293-295: I suggest re-phrasing this sentence as it is slightly difficult to follow it, e.g., 'positive development between' is not an understandable term.

-Line 296: I suggest you change 'being' to 'which are'.

-Line 307: Suggest you change 'fly in' to 'swing by' or 'pop over'.

-Line 308: ' In those moments', what 'moments' are the authors referring to? It is unclear.

-Lines 315-316: 'A study in Canada on GPs’ experiences with prescribing pharmacists', please change to 'A Canadian study on GPs' experiences of prescribing pharmacists'.

-Lines 316-317: '(both community pharmacists and NDPs, so-called team pharmacists)', so, was this combination of community pharmacists and NDPs called 'team pharmacists' in that study? If so, change this section to '(called as 'team pharmacists' in that study and involving both community pharmacists and NDPs)'.

-Line 318: Please remove square brackets, so to look like 'collaboration... All'.

-Line 320: 'So, besides the need for proximity, this study...', which study are you referring to? Reference 35 or your current study? It is not currently clear.

-Lines 321-326: it is usually not a good practice having quoted arguments over multiple sentences. Try to indicate in each of these sentences that arguments originate from ref 15, e.g. by saying 'That same UK study reported that "In practices where the pharmacy team ..."'.

-Lines 323 and 325: please remove 'PG5' and 'GP7' as readers might confuse those participants in that study with your participants.

-Lines 331-333: 'The fact that collaboration with the NDP-like pharmacists was perceived easier than with community pharmacists by the Canadian GPs could imply that alignment of professional identities already had started between them, too', this sentence is really confusing, e.g., 'was perceived'-by whom, where and when?; 'alignment of professional identities has started between them', between whom, where and when? Which studies are you talking about? I strongly suggest you re-phrase this sentence.

- Lines 333-336: 'Also in our study, a difference between collaborating with NDPs and community pharmacists was perceived by GPs; so, we hypothesise that despite (often) proximity between GPs and community pharmacists and (often) knowing each other, the community pharmacists and GPs may not have aligned their professional identities, whereas NDPs and GPs have', this sentence is unclear and does not convey your intended meaning. Suggest you re-phrase to: 'GPs in our study reported a difference between collaboration with NDPs and collaboration with community pharmacists, so, we hypothesise that despite (frequent) proximity between GPs and community pharmacists and (frequent) mutual relationships, community pharmacists and GPs may not have aligned their professional identities, whereas NDPs and GPs may have had'.

-Lines 337-338: Please change to 'Over time, in the process of aligning identities, NDPs the GPs may start to reconsider responsibilities'.

-Lines 338 and 335: 'integral care provided to patients', this is a slightly bizarre phrase. Please explain what do you mean or consider re-phrasing.

-Lines 339-340: Suggest you change to 'On the one hand, GPs want to be responsible for and in control of patient care', or to something along those lines.

-Lines 343-344: “[The NDP] did not need to seek approval prior to prescribing whereas community pharmacists should.”, is there a way you can clarify what problem this was actually generating? Was it in relation to time-constraints, workload etc., for example?

-Lines 346-347: 'we think that talking to and discussing with one another', it is unclear to whom this refers. Possibly change to 'we think that mutual interactions between GPs and NDPs'.

-Lines 347-348: 'how to relate to such reconsiderations and changes', it is unclear what you mean with 'reconsiderations' and 'changes'. Please make it clear for readers.

-Lines 551-552: Totally not clear what 'standardise' means in here and how this related to what you did in the current study. Please make it clear to readers. I also suggest you change 'regarded' to 'revealed' or 'explored'.

-Line 354: 'successful implementation of the model in other practices with their own context', which 'model' are you referring to? Also, what does 'with their own context ' mean? Do you mean 'within their own circumstances'?

-Line 355: Some limitations need to be considered too, suggest you change to 'This study had several limitations'.

-Lines 357-358: 'During this time, five of the nine practices the NDPs continued working after the intervention period ended while in the other practices the NDPs stopped, bringing different stories to the fore', suggest you change to 'During this time, in five of the nine practices the NDPs continued working after the intervention period ended, while in the rest of the practices the NDPs stopped working, bringing different priorities/experiences/problems to the fore'.

-Line 359: 'contrast between the interviews', 'contrast' does not convey your intended meaning. Do you mean opposition to NDPs amongst the interviewees?

-Line 361: Change 'contrast' to 'account for' or 'reflect' or 'mirror'.

-Line 363: remove 'had', so that it reads 'would have added'.

-Lines 361-363: Stemming from your reference to other stakeholders here, I suggest that somewhere, probably in the Introduction section so to better highlight the gap in knowledge you are trying to address (i.e., what works well and why as experienced by GPs), you account for work that has explored what works well and what does not with UK general practice-based pharmacists. There is quite a volume of available UK literature. Then, you can move on by stating differences with Dutch healthcare system and why international literature might not be applicable, hence the need for your work.

-Line 369: 'the needed means' does not mean anything. Please consider re-phrasing to a more understandable phrase.

-Line 370: What does 'sustainability of impact' mean? Wouldn't just saying 'impact' suffice?

-Lines 370-373: Sentence is really large and meaning is lost, please consider splitting it into two smaller sentences.

-Line 374: 'the social factors that we have identified', please explain what do you mean with 'social factors'.

-Line 374: 'should be focus of research', suggest you change to 'should be the focus of future research'.

-Lines 374-375: 'The need for adequate financing has been reported by GPs in earlier research on comparable models. [29] ', this sentence is totally unclear. 'Financing' on what and where? What does 'comparable models' mean? Strongly suggest that you re-phrase this sentence.

-Lines 375-376: I do not understand at all what 'financing' has to do with 'why and how the model works'. 'Funding' on what? Do you mean 'funding' of research? Apologies, as a reader, I struggle to understand this argument.

-Line 378: Please define 'interprofessional training', it is not a self-explanatory term for readers.

-Line 381: Please define 'workplace learning', not a self-explanatory term.

Conclusion:

-Lines 385-386: I suggest you change 'but also via professional identities alignment of NDP and GP' to 'but also via NDPs and GPs aligning their professional identities'.

-Lines 387-389: 'To induce these mechanisms when broader implementing the interprofessional model with NDPs, GPs need to acknowledge that the need for improvement of the quality of pharmaceutical care is an interprofessional endeavour', there are some problems with this sentence. First of all, explain what 'these mechanisms' refer to. Second, re-phrase 'when broader implementing the interprofessional model with NDPs' to 'when broader implementation of the interprofessional model with NDPs in general practices is sought'. Third, I am not sure what 'interprofessional endeavour' means, please explain/define.

-Line 391: change 'pharmacotherapy for' to 'pharmacotherapy to'.

Reviewer 2

Submitted: 27 Sep 2023

Feedback for the author(s)

It was my absolute pleasure to review this paper submitted to BMC Health Services Research. Th study describes a realist evaluation of non-dispensing pharmacists (NDPs) working in GP surgeries in the Netherlands. This is an important piece of research with far-reaching implications for similar models of practice elsewhere in the world. The term NDP is not used in the UK, however it is a good way of describing this role in a consistent way (rather than GP pharmacist, primary care pharmacist etc.). Given the contextual issues that depend on the success of such schemes, I can see the value in using this interview schedule applied to similar models of care in different settings and different countries for comparison. Nevertheless some of the key findings are significant and generalisable in terms of aligning professional identity and the interprofessional learning required to optimise this new role. The study adopts a robust approach, drawing on implementation science methodology and the data have been synthesised ably.

I recommend that the paper is published pending a few very minor changes, as detailed below:

1. Methods - put ER in full - it may have been presented earlier in the paper, but if so, I missed it.

2. Results - provide a very brief overview of the key findings / headlines in the first paragraph before describing the results in more detail.

3. Discussion - what are the implications for pharmacists working in GP surgeries who have prescribing qualifications? These roles are on the rise in the UK therefore some reference to how NDPs could also provide this function or the need for further research on this specific role would be needed.

Reviews to Version 2:

Reviewer 1

Submitted: 5 Feb 2024

Feedback for the author(s)

The authors have introduced all requested changes. I believe that the manuscript is now ready for publication and I look forward to seeing it published.

Reviewer 3

Submitted: 27 Jan 2024

Feedback for the author(s)

Thank you for the opportunity to review a revised version of this interesting paper. This is an important piece of work and is timely in the context of the expanding role of non-dispensing clinical pharmacists in general practice. The authors have addressed the reviewers' comments and I appreciate the detailed responses of the authors. I only have minor comments with the manuscript in its current form. These are stated below:

- Please add the table of baseline characteristics of GPs to the results section as this table is mentioned in the response letter, however, it is not cited at all within main text. The results would benefit from detail about the demographics of the participants.

- Please change the ‘topic list' to 'topic guide' in the Online Supplement S1 as this was changed in the manuscript.

Methods-Line 168: Do you mean ‘negative or positive’ instead of ‘negative of positive ‘?
